# Problematic internet use and safety behavior: The moderating role of safety climate

**DOI:** 10.1371/journal.pone.0279767

**Published:** 2022-12-30

**Authors:** Fakhradin Ghasemi, Hamed Aghaei, Asghar Nikravesh

**Affiliations:** 1 Department of Occupational Health and Safety Engineering, Abadan University of Medical Sciences, Abadan, Iran; 2 Department of Occupational Health Engineering, School of Health, Arak University of Medical Sciences, Arak, Iran; 3 Department of HSE, Golgohar Mining and Industrial Company, Kerman, Iran; Kaohsiung Medical University, TAIWAN

## Abstract

Problematic internet use (PIU) can cause mental and physical harm to individuals and may be an emerging factor contributing to unsafe work behavior. In this study, the relationship between PIU and safety behavior was investigated. Moreover, it was hypothesized that safety climate can moderate the relationship between PIU and safety behavior. Participants were employees from a mining industry in Iran. Three validated questionnaires were distributed to gather the required data regarding PIU, safety climate, and safety behavior. Hierarchical regression analysis was used to assess the moderating effect of safety climate on the relationship between PIU and safety behavior. The size of moderation effect was assessed using f^2^ index. Four hundred eighty-five employees participated in this study. The results demonstrated a negative relationship between PIU and safety behavior. The moderating effect of safety climate on the relationship between PIU and safety behavior was supported. The f^2^ index was 0.027 which demonstrates a small moderation effect. In conclusion, PIU has a significant negative effect on the safety behavior of employees. Providing a strong safety climate can be a long-term solution for reducing the negative effect of PIU on safety behavior.

## Introduction

Safety behavior refers to those behaviors which are in line with safety standards, rules, norms, and policies of the organization. Lack of safety behavior can make organizations prone to accidents [1, 2]. The contribution of unsafe work behaviors to most industrial accidents has been asserted by many studies [1, 3, 4]. There are many factors causing employees to engage in unsafe work behavior. These factors have been discussed by many studies and several remedies also have been developed. However, the ever-changing world can create new factors affecting the safety behavior of employees at workplaces. One of these factors is the omnipresence of the Internet and digital devices such as laptops, mobile phones, and tablets [5]. Nowadays, most people have continuous access to the internet and online information. The attracting nature of such information can lead to “problematic internet use (PIU)”.

PIU is defined as “the intemperate utilize of the Internet that disrupts or harms the individual” [5]. Organizations confront productivity losses as a result of workers’ association in counterproductive behaviors, such as PIU. Some previous researches showed that more than half of Internet-enabled workers go online for individual purposes at an average rate of three hours per week [6] and some recent researches show that the number of hours has increased to almost 1h per day [7]. Other studies illustrated that workers go online for more than one hour for non-work-related activities at work per day [8]. Between 60% and 80% of workers use the internet for non-work-related activities, leading to around 30% to 40% decrease in productivity [9]. It is estimated that Internet usage for non-work-related activities in the workplace in the United States costs approximately $85 billion yearly [10]. Internet usage for non-work-related activities in the workplace not only results in poor productivity but also slacks network security and makes the organization to cyber-attacks [11]. In addition to the aforementioned issues, PIU can lead to adverse physical and mental outcomes as well. Depressive symptoms [12], mental health problems [13], social fears [14], irritability and sleep problems [15] low physical [16], risk-taking behaviors [17], and poor academic performance [18] are some of these consequences reported by previous studies. PIU is associated with a decrease in a wide range of neuropsychological functions [19].

Studies demonstrated that some cognitive failures are related to PIU [20]. Hadlington [21] showed a connection between higher PIU use and self-reported cognitive failures in daily life. Firth et al. [22] investigated the effect of the internet on attentional capacities, memory processes, and social cognition. They explained that the constant access and use of the Internet creates a tendency among individuals to use divided attention, making it difficult for them to perform tasks that require sustained attention. Moreover, excessive reliance on the internet and online information makes individuals reluctant to save information in their long-term memory and also disrupts memory retrieval of information [22–24]. Altogether, these explanations indicate that PIU can lead to cognitive failures which have been well-documented in previous studies [20, 21]. On the other hand, cognitive failures have been known to play a major role in engaging in unsafe work behavior [25]. Furthermore, the constant access to online information may change the perception of individuals regarding their knowledge by making indistinct the line between one’s capabilities and her/his digital devices [22, 26]. The phenomenon can develop an illusion of “greater than actual knowledge” among individuals [22]. This state can be very dangerous in occupational settings where plenty of hazards are present. For example, an employee with the illusion of knowing everything about a process or task may ignore the available safety procedures (a type of unsafe work behavior). Such ignorance can result in unsafe behaviors and thereby industrial accidents. Consequently, and based on these explanations, it can be hypothesized that PIU can cause unsafe behavior in occupational settings.

Safety climate is the shared perception of employees toward safety within the organization [27]. Safety climate is one of the most important factors affecting safety behavior at workplaces [1, 28–30]. A wide range of factors has the capability of affecting safety behavior, safety climate, as a strong moderator, can attenuate the negative effect of such factors on safety behavior. According to Hussain et al. [31], the effect of socio-cognitive factors on the risky driving behavior of truck drivers is moderated by safety climate. The negative effects of job insecurity on safety outcomes such as safety behavior and occupational injuries are alleviated by a strong positive safety climate [32]. Zhou and Jiang [33] demonstrated that the relationship between leader-member exchange is positive when there is a strong safety climate in the workplace, while the relationship is negative when safety climate is poor. In the same vein, safety climate moderates the effect of stress responses on safety behavior [34]. Moreover, providing a strong positive safety climate has some additional benefits. For example, it makes less severe the negative effect of staffing inadequacy and unpleasant work conditions on occupational injuries among nurses [35]. PIU can be an emerging cause of unsafe behavior at workplaces. So far, to the best of our knowledge, no study investigates the role played by safety climate on this relationship, so it can be hypothesized that safety climate can moderate the relationship between PIU and safety behavior.

Accordingly, the present study was conducted to assess the effect of PIU on safety behavior. Moreover, the study aimed at assessing the moderating effect of safety climate on the relationship between PIU and safety behavior. Accordingly, the study had two hypotheses:

Hypothesis 1: PIU is negatively associated with safety behavior,
Hypothesis 2: the effect of PIU on safety behavior is moderated by safety climate.

**Fig 1:**
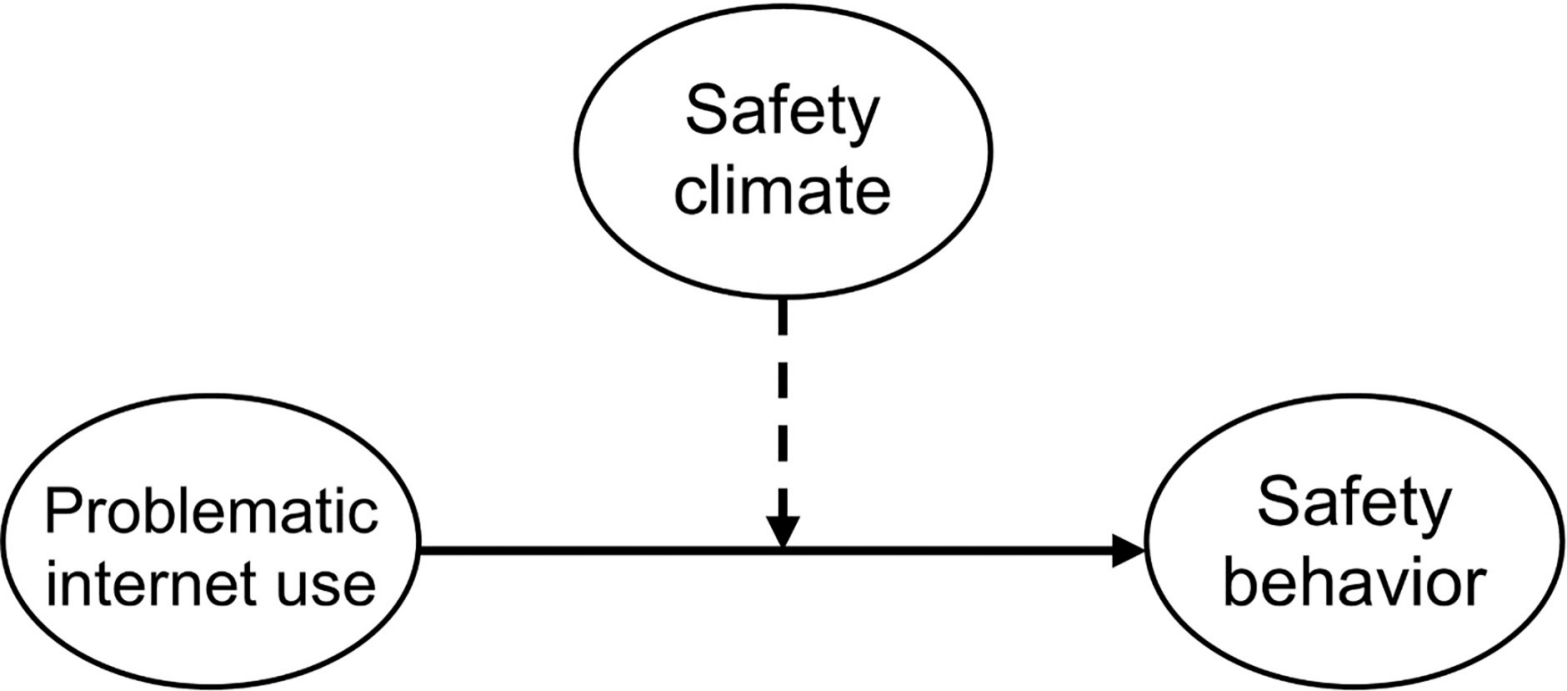
The hypothetical model.

Demonstrated in Fig 1 is the hypothetical model of the present study.

## Material and methods

### Ethical statement

The participation was voluntary and participants were assured that their information remain confidential. First, objectives and methodology of the study were verbally explained to participants and they were verbally asked about their willingness to participate in the study, the questionnaires were distributed among those who agreed to participate. Moreover, the first item of the questionnaires asked about this issue again; "would you like to participate in this study?" and participants that answered yes were included in the study. The study protocol was approved by the ethics committee of Abadan university of medical sciences (ethic code: IR.ABADANUMS.REC.1400.079).

### Participants

There are many active underground and surface mines in Iran. Based on some statistics, Iran is among 15 major mineral-rich countries. Iron ore, copper, zinc, gypsum, molybdenum, and antimony are some important minerals extracted in Iranian mines. Although the industry has not been fully developed, at least half-billion employees are working in this sector directly or indirectly. In this study, employees from an open-pit mining industry located in the east of Iran were invited to participate in the study. The table from Krejcie and Morgan [36] was used to determine the required sample size. Given the total number of nine thousand employees in the company, the sample size was determined to be 368. However, the response rate is rarely 100% and we had to invite more participants to the study. Consequently and to guarantee the required sample size, the determined sample size was conservatively multiplied by 2, so 736 questionnaires were randomly distributed among employees.

### Data gathering tool

Standard and validated questionnaires were used to gather the required data. A 6-item scale was developed by Siciliano et al. [37] to measure PIU. The scale was later modified by Abubakar and Al-Zyoud [5] to be usable in occupational settings. In this study, both tools were adopted to develop a Persian scale appropriate for the Iranian population. Content validity index (CVI) and content validity ratio (CVR) of all items were assessed before application [38, 39]. “I feel nervous and restless when I am offline and these feelings are disappeared when I go back online”, “I think that I use mobile phone and internet more than what I really need”, “sometimes I forget or neglect safety considerations because of being busy with mobile phone and internet”, and “I prefer to use mobile phone and internet instead of going out with my friends or exercising” are some items used to measure PIU. All items were responded using a 5-point Likert scale ranging from 1 (totally disagree) to 5 (totally agree).

A 7-item scale from Ghasemi et al. [28] was used to assess perceived safety climate (acceptable content validity and Cronbach’s alpha coefficient = 0.870). “In my workplace, safety is as important as production” and “Managers actively participate in designing and implementing safety programs” are some items used for measuring safety climate [28]. All items were responded using a 5-point Likert scale ranging from 1 (totally disagree) to 5 (totally agree).

A 6-item scale was used for measuring safety behavior. The items were adopted from Neal and Griffin [3] which were translated and validated into Persian by Mahdinia et al. [40]. The scale encompasses both safety participation and safety compliance aspects of safety behavior and demonstrates acceptable reliability and validity (Cronbach’s alpha coefficient = 0.8 and acceptable content validity).

### Data analysis

First, the construct validity of the variables was assessed. To do so, a measurement model was built and the goodness of fit of the model was assessed using absolute and comparative indices such as RMSEA, chi-square/df, NFI, RFI, GFI, CFI, and so on. RMSEA lower than 0.08, chi-square/df lower than 3, and comparative indices higher than 0.9 were considered to be acceptable [28, 41, 42]. Convergent and discriminant validity are two main aspects to be investigated during construct validity assessment.

The convergent validity of variables was investigated based on standardized factor loadings (SFLs) and average variance extracted (AVE). SFLs higher than 0.3 and AVE higher than 0.5 were considered to be acceptable, as recommended by Bagozzi and Yi [43]. The discriminant validity of variables was evaluated by comparing the square root of AVE with correlation coefficients among variables. The discriminant validity is confirmed if the AVE square root of a variable is higher than its correlations with other variables.

Hierarchical regression analysis in accordance with the procedure explained by Helm and Mark [44] was conducted to assess the moderating effect of safety climate on the relationship between PIU and safety behavior. This approach is one of the most popular methodologies utilized for the assessment of moderation effect [44]. In this respect, two regression models were constructed and examined. The first model was the basic model in which PIU and safety climate were predictors and safety behavior was the dependent variable (Eq 1). The second model is called the interaction model in which PIU, safety climate, and the interaction term (i.e. PIU × safety climate) are predictors, and safety behavior is the dependent variable (Eq 2). The moderation effect is supported if the effect of the interaction term is significant [44].


$$Basic\;model:\;SB=b_{0}+SC.b_{1}+{PIU.b}_{2}$$ (1)

$$Interaction\;model:\;SB=b_{0}+SC.b_{1}+{PIU.b}_{2}+SC\times PIU.b_{3}$$ (2)

Where SB safety behavior, SC = safety climate, PIU = problematic internet use, and b_1_, b_2_, and b_3_ are regression weights.

Visualization of the moderation effect can provide a deeper insight on how the moderation works. For this purpose, the procedure recommended by Hayes [45] was employed. In this regard, two new equations (simple slope lines) were generated from the interaction model formula by inserting certain values of the moderator into the equation, i.e. safety climate = mean + SD and safety climate = mean–SD. The effect of moderation variable would be visualized when these two slope lines are plotted for various values of PIU.

Moreover, the size of moderation effect was calculated using Eq 3 [46]:

$$f^{2}=\frac{R_{i}^{2}-R_{b}^{2}}{1-R_{i}^{2}}$$ (3)

Where f^2^ = the size of moderation effect, $R_{i}^{2}$ = R^2^ of the interaction model, and $R_{b}^{2}$ = R^2^ of the basic model. The size of moderation effect is negligible when its value is lower than 0.002.

## Results

A total number of 485 employees from a mining industry participated in this study (response rate = 66%). All participants were male, most of them were married (84.1%), most of them were aged between 31 and 40 years old (61.4%), and most of them had 6–10 years of experience (35.7%). Detailed demographic characteristics of the participants are demonstrated in Table 1. They were working in various units of the mining company, including extraction, processing mineral concentrates, and pelletizing units.

**Table 1:** Demographic characteristics of participants (n = 485).

Variable	Number (%)
Age (year)	
= < 30	115 (23.7%)
31–40	298 (61.4%)
41–50	63 (13.0%)
> 50	9 (1.9%)
Experience (years)	
<1	56 (11.5%)
1–5	159 (32.8%)
6–10	173 (35.7%)
11–15	70 (14.4%)
>15	27 (5.6%)
Marital status	
Single	77 (15.9%)
Married	408 (84.1%)

### Construct validity

**Fig 2:**
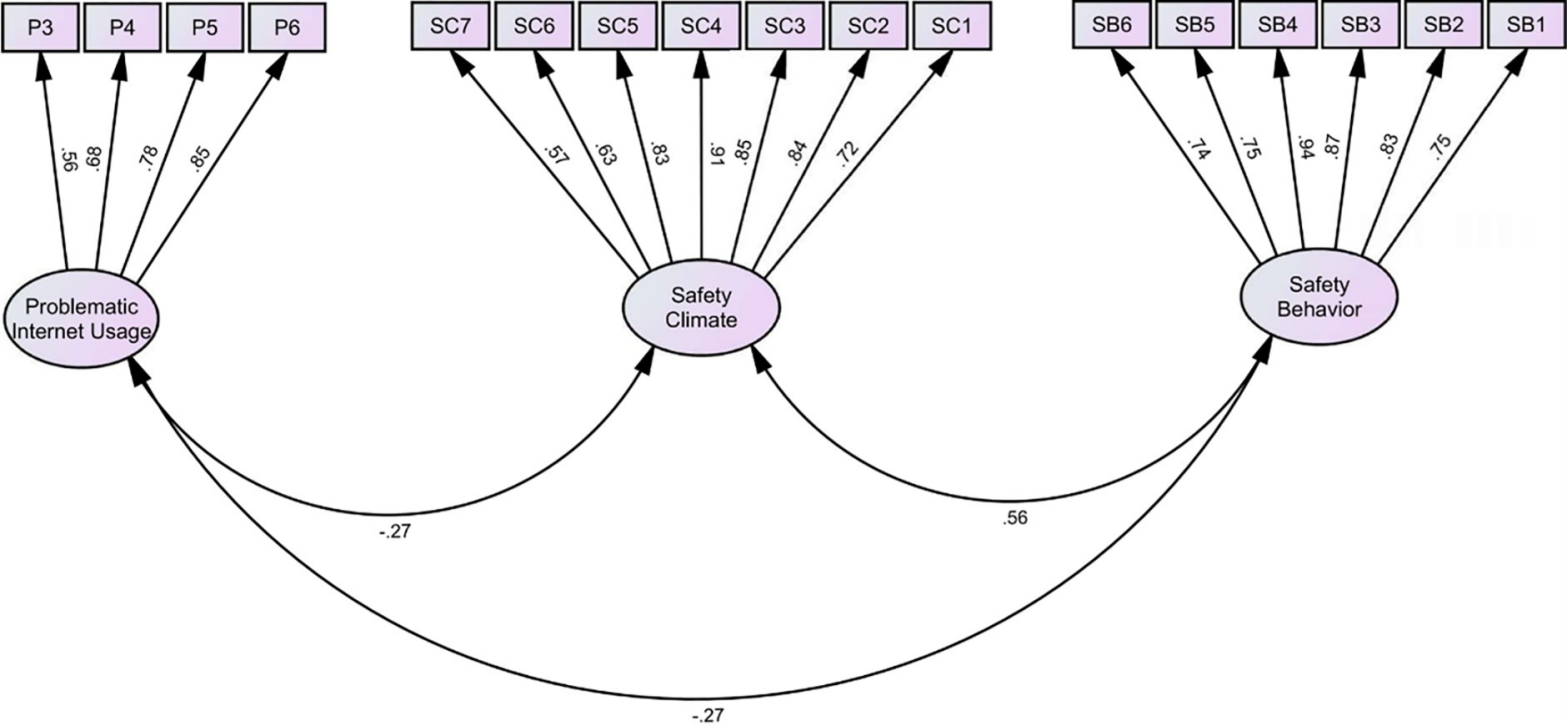
The measurement model.

Confirmatory factor analysis was performed to assess the construct validity of variables. The CFA model is depicted in Fig 2 and it was satisfactory in terms of both absolute and comparative model fit indices: Chie-square/df = 2.47, RMSEA = 0.055, GFI = 0.939, NFI = 0.954, RFI = 0.942, IFI = 0.972, TLI = 0.964, and CFI = 0.972. SFL of each item on the related construct and AVE were two criteria based on which convergent validity was evaluated. Two items related to the PIU had an SFL lower than 0.3, so they were excluded. As demonstrated in Table 2, all SFLs are higher than 0.3 and all AVEs are higher than 0.5, so the convergent validity of the constructs is satisfactory. Discriminant validity was assessed by comparing AVE root square of variables with their correlation coefficients. As the AVE root square of all variables was higher than correlation coefficients among them, the discriminant validity of variables was also supported.

**Table 2:** Construct validity of problematic internet use (PIU), safety climate, and safety behavior.

Variable	Item	Standardized factor loading (SFL)	AVE ($\sqrt{AVE}$)	Cronbach’s α
Problematic internet use	P1	-	0.53 (0.73)	0.81
	P2	-		
	P3	0.56		
	P4	0.68		
	P5	0.78		
	P6	0.85		
Safety Climate	SC1	0.72	0.60 (0.77)	0.91
	SC2	0.84		
	SC3	0.85		
	SC4	0.91		
	SC5	0.83		
	SC6	0.63		
	SC7	0.57		
Safety Behavior	SB1	0.75	0.67 (0.82)	0.92
	SB2	0.83		
	SB3	0.87		
	SB4	0.94		
	SB5	0.75		
	SB6	0.74		

### Bivariate analysis

The results of bivariate analyses are presented in Table 3. Based on the type of variables, Pearson or Spearman coefficients were calculated. As evident, personal characteristics such as age, experience, and marital status had no significant correlation with PIU, safety climate, and safety behavior. PIU was negatively related to both safety behavior and safety climate. As expected, a significant positive relationship was also observed between safety climate and safety behavior.

**Table 3:** Correlations among variables investigated in this study.

	PIU	SC	SB	Age	Experience
PIU	1				
SC	-0.246**	1			
SB	-0.230**	0.547**	1		
Age	0.050	-0.008	0.003	1	
Experience	-0.100*	0.047	0.004	0.412**	1
Marriage	0.002	0.014	-0.013	0.329**	0.293**

Note: PIU: Problematic internet use; SC: Safety climate; SB: Safety behavior

**p < 0.01

*p < 0.05.

### Moderation analysis

Hierarchical regression analysis was employed to assess the moderation effect of safety climate on the relationship between PIU and safety behavior and the results are presented in Table 4. In the basic model, both PIU and safety climate had a significant effect on safety behavior. In the interaction model, the interaction term also had a significant effect on safety behavior. Accordingly, the main hypothesis of the study was supported so that safety climate moderated the effect of PIU on safety behavior. The f^2^ index was calculated to determine the size of moderation effect. The value of this index was 0.027 which demonstrates a small effect size.

**Table 4:** Basic and interaction models for assessing the moderation effect of safety climate on the relationship between problematic internet use and safety behavior.

Model	predictor	B	SE	Beta	t	p	R	R^2^	f^2^
Basic	PIU	-0.136	0.052	-0.102	-2.607	<0.01	0.556	0.309	0.027
	SC	0.375	0.028	0.522	13.372	<0.01			
Interaction	PIU	-0.766	0.182	-0.575	-4.201	<0.01	0.572	0.327	
	SC	0.146	0.069	0.203	2.095	0.037			
	PIU×SC	0.031	0.008	0.520	3.603	<0.01			

Note: PIU: Problematic internet use; SC: Safety climate

For a deeper insight into the moderation effect of safety climate on the relationship between PIU and safety behavior, two simple slope lines were generated from the interaction model formula as follows:

SB=19.031+(−0.766×PIU)+(0.146×SC)+(0.031×PIU×SC) (4)

$$For\;SC=\bar{x}-SD,\;SB=21.350+(-0.274\times PIU)$$ (5)

$$For\;SC=\bar{x}+SD,\;SB=22.759+(0.026\times PIU)$$ (6)

**Fig 3:**
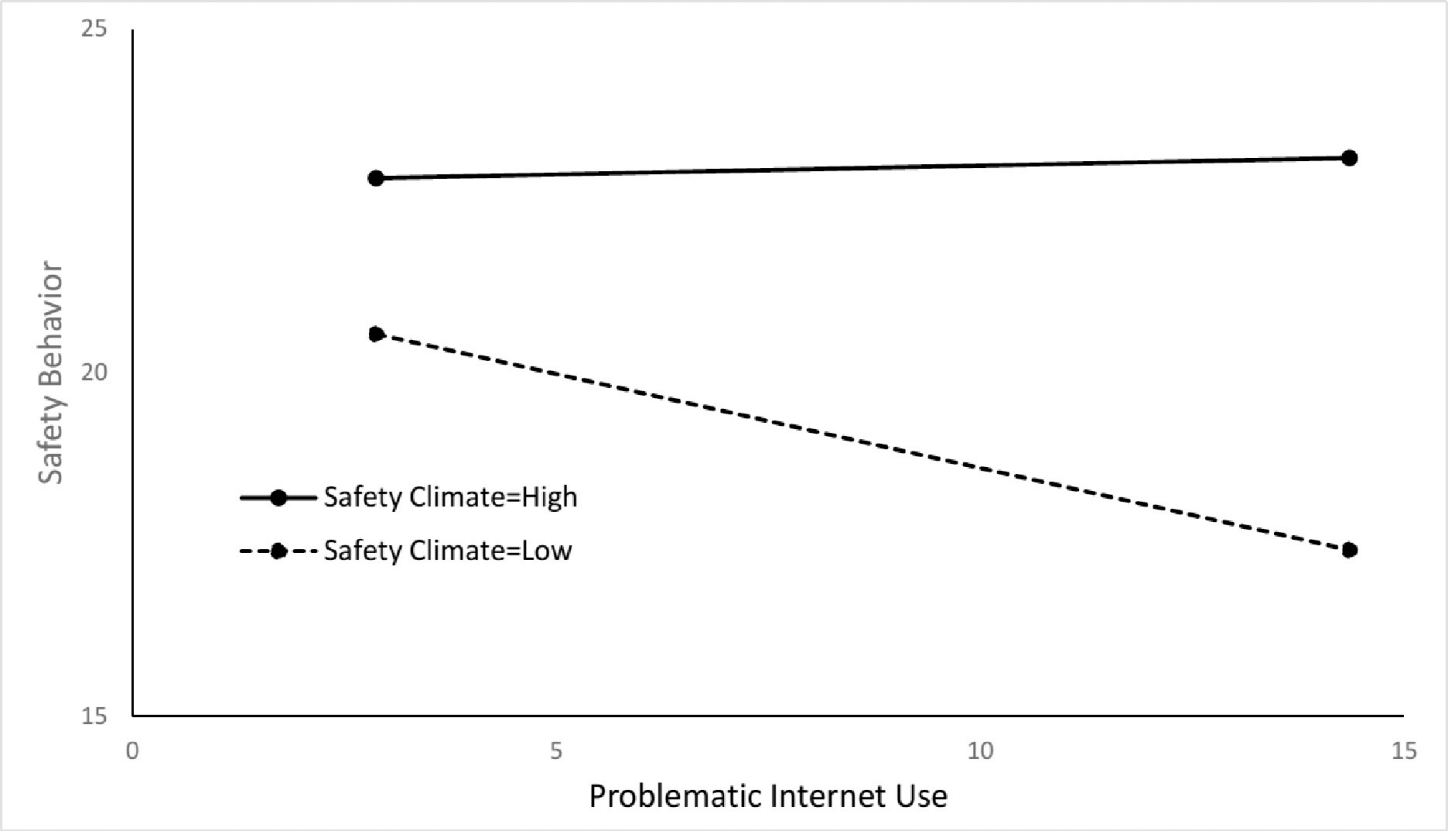
The slope analysis of the moderation effect.

These lines are depicted in Fig 3 based various values of PIU. When safety climate was high (i.e. Mean + SD), the slope of regression line was very close to zero, indicating that PIU lost its effect on safety behavior in such situations. In contrast, when safety climate was low (i.e. Mean–SD), safety behavior was drastically decreased with the increase of PIU, suggesting that the lack of a positive safety climate made the negative effect of PIU on safety behavior stronger.

## Discussion

Nowadays, as using the internet is inevitable for carrying out many occupational and non-occupational activities, PIU can be an emerging cause of unsafe work behavior. The addictive potential of the internet is progressively increased by reinforcing attention-capturing characteristics of websites and apps without sufficient consideration of its probable effects on human health, safety, and well-being [22, 47]. Although the relationship between PIU and accidents, particularly car and truck collisions, has been the subject of several studies [48, 49], studies investigating the relationship between PIU and safety behavior are rare. Meanwhile, the effect of PIU and cyber-loafing on job performance has been shown to be inconsistent across literature [50]. Accordingly, in this study, the effect of PIU on safety behavior and also the moderating effect of safety climate on this relationship were investigated.

A negative significant relationship between PIU and safety behavior was identified in this study. Similarly, Abubakar and Al-Zyoud [5] investigated the relationship between PIU and safety behavior in the marine sector and found a significant relationship between these two variables. Furthermore, Oktan [51] reported a positive significant relationship between PIU and risk taking behavior among high school students and Guo et al. [52] found that PIU can increase the risk of suicidal behavior. As mentioned before, the effect of PIU on safety behavior can be mediated by cognitive failures. Moreover, PIU is associated with mental problems such as depressive symptoms, stress, and burnout [53]. As some of these problems are associated with poor safety behavior [54], they might be able to mediate the relationship between PIU and safety behavior at workplaces. However, there is a need for more researches in this area.

This study recommended safety climate as a way through which the negative effect of PIU on safety behavior can be alleviated. The analyses demonstrated the moderation effect of safety climate on this relationship so that safety climate can relieve the negative effect of PIU on safety behavior. This finding emphasizes that providing a positive safety climate is effective in reducing the effect of newly-emerged issues such as PIU on safety behavior. The finding is in line with numerous previous studies emphasizing the buffering capacity of a positive safety climate in workplaces [28, 33]. Safety climate is the shared perception and attitude of employees toward safety. There are several factors constituting safety climate including management commitment to safety, supportive environment for safety, safety training, safety procedures, and so on [55, 56]. Improving these dimensions, particularly management commitment to safety, is necessary for creating a safety climate at workplaces. Once such a positive environment is created, the negative impact of PIU on safety behavior can be expected to decrease. In the same vain, Abubakar and Al-Zyoud [5] recommended time autonomy as a factor moderating the effect of PIU on safety behavior.

PIU is a huge problem in both personal life and workplaces because it can result in many undesirable outcomes [5, 57]. Moreover, all problems created by PIU have yet to be identified mainly because the pace of technological progress is much faster than researches conducted in this field [57]. PIU should be managed properly nonetheless. Some organizations inhibit the use of mobile and the internet for personal purposes in workplaces, however, organizations have no control over the off-the-job activities of their employees so they may still be affected by the bad consequences of PIU. Cognitive-behavioral therapy has been recommended for treating PIU [57] while increasing the knowledge of individuals regarding the adverse side effects of PIU may be a preventive strategy. Moreover, Davis et al. [58] developed a pre-employment tool for singling out people who are prone to abuse the internet in the workplaces. This tool may be used for selecting and employing individuals with less proneness toward PIU. Training regarding the adverse effects of Internet addiction is another countermeasure in this respect [59].

Overall, studies investigating the effect of PIU on safety outcomes in workplaces are rare and more studies are needed in this respect. There are several factors that are able to mediate the effect of PIU on safety behavior, including poor sleep quality and mental disorders stem from PIU. Moreover, the effect of PIU on injuries and occupational accidents needs more researches.

## Conclusion

PIU can negatively affect safety behavior so employers should pay special attention to this subject. Safety climate can attenuate the negative effect of PIU on safety behavior. Strategies for reducing PIU and enhancing safety climate are required to improve the safety behavior of employees.

## Supporting information

S1 File Data used in the study. (DOCX)

## References

[1] Ghasemi F, Aghaei H, Askaripoor T, Ghamari F. Analysis of occupational accidents among nurses working in hospitals based on safety climate and safety performance: A Bayesian Network analysis. Int J Occup Saf Ergon. 2022;28: 440–446. doi: 10.1080/10803548.2020.1768759 32508274

[2] Mahdinia M, Mohammadfam I, Soltanzadeh A, Aliabadi MM, Aghaei H. A fuzzy Bayesian network DEMATEL model for predicting safety behavior. Int J Occup Saf Ergon. 2021; 1–8. doi: 10.1080/10803548.2021.2015741 34898390

[3] Neal A, Griffin MA. A study of the lagged relationships among safety climate, safety motivation, safety behavior, and accidents at the individual and group levels. J Appl Psychol. 2006;91: 946–953. doi: 10.1037/0021-9010.91.4.946 16834517

[4] Aghaei H, Mirzaei M, Id A, Mollabahrami F, Najafi K. Human reliability analysis in de-energization of power line using HEART in the context of Z-numbers. PLoS One. 2021;16: e0253827. doi: 10.1371/journal.pone.0253827 34197502 PMC8248607

[5] Mohammed Abubakar A, Al-zyoud MF. Problematic Internet usage and safety behavior: Does time autonomy matter? Telemat Informatics. 2021;56: 101501. doi: 10.1016/j.tele.2020.101501

[6] Yellowlees PM, Marks S. Problematic Internet use or Internet addiction? Comput Human Behav. 2007;23: 1447–1453. doi: 10.1016/j.chb.2005.05.004

[7] Tseng VWS, Lee ML, Denoue L, Avrahami D. Overcoming Distractions during Transitions from Break to Work using a Conversational Website-Blocking System. Conf Hum Factors Comput Syst—Proc. 2019. doi: 10.1145/3290605.3300697

[8] Shrivastava A, Sharma MK, Marimuthu P. Internet addiction at workplace and it implication for workers life style: Exploration from Southern India. Asian J Psychiatr. 2018;32: 151–155. doi: 10.1016/j.ajp.2017.11.014 29275219

[9] Nazareth DL, Choi J. A system dynamics model for information security management. Inf Manag. 2015;52: 123–134. doi: 10.1016/j.im.2014.10.009

[10] Son JY, Park J. Procedural justice to enhance compliance with non-work-related computing (NWRC) rules: Its determinants and interaction with privacy concerns. Int J Inf Manage. 2016;36: 309–321. doi: 10.1016/j.ijinfomgt.2015.12.005

[11] Leuprecht C, Skillicorn DB, Tait VE. Beyond the Castle Model of cyber-risk and cyber-security. Gov Inf Q. 2016;33: 250–257. doi: 10.1016/j.giq.2016.01.012

[12] Romer D, Bagdasarov Z, More E. Older versus newer media and the well-being of united states youth: Results from a national longitudinal panel. J Adolesc Heal. 2013;52: 613–619. doi: 10.1016/j.jadohealth.2012.11.012 23375827

[13] Zhou N, Cao H, Liu F, Wu L, Liang Y, Xu J, et al. A four-wave, cross-lagged model of problematic internet use and mental health among chinese college students: Disaggregation of within-person and between-person effects. Dev Psychol. 2020;56: 1009–1021. doi: 10.1037/dev0000907 32105117

[14] Lee BW, Stapinski LA. Seeking safety on the internet: Relationship between social anxiety and problematic internet use. J Anxiety Disord. 2012;26: 197–205. doi: 10.1016/j.janxdis.2011.11.001 22169053

[15] Zhou Y, Lin FC, Du YS, Qin L Di, Zhao ZM, Xu JR, et al. Gray matter abnormalities in internet addiction: A voxel-based morphometry study. Eur J Radiol. 2011;79: 92–95. doi: 10.1016/j.ejrad.2009.10.025 19926237

[16] Rosen LD, Lim AF, Felt J, Carrier LM, Cheever NA, Lara-Ruiz JM, et al. Media and technology use predicts ill-being among children, preteens and teenagers independent of the negative health impacts of exercise and eating habits. Comput Human Behav. 2014;35: 364–375. doi: 10.1016/j.chb.2014.01.036 25717216 PMC4338000

[17] Dou K, Wang LX, Li J Bin, Wang GD, Li YY, Huang YT. Mobile phone addiction and risk-taking behavior among chinese adolescents: A moderated mediation model. Int J Environ Res Public Health. 2020;17: 1–13. doi: 10.3390/ijerph17155472 32751334 PMC7432004

[18] Zhang Y, Qin X, Ren P. Adolescents’ academic engagement mediates the association between Internet addiction and academic achievement: The moderating effect of classroom achievement norm. Comput Human Behav. 2018;89: 299–307. doi: 10.1016/j.chb.2018.08.018

[19] Hasenhütl G. The World Beyond Your Head: On Becoming an Individual in an Age of Distraction. J Mod Cr. 2018;11: 287–291. doi: 10.1080/17496772.2018.1538631

[20] Ioannidis K, Hook R, Goudriaan AE, Vlies S, Fineberg NA, Grant JE, et al. Cognitive deficits in problematic internet use: meta-analysis of 40 studies. Br J Psychiatry. 2019;215: 639–646. doi: 10.1192/bjp.2019.3 30784392 PMC6949138

[21] Hadlington LJ. Cognitive failures in daily life: Exploring the link with Internet addiction and problematic mobile phone use. Comput Human Behav. 2015;51: 75–81. doi: 10.1016/j.chb.2015.04.036

[22] Firth J, Torous J, Stubbs B, Firth JA, Steiner GZ, Smith L, et al. The “online brain”: how the Internet may be changing our cognition. World Psychiatry. 2019;18: 236–237. doi: 10.1002/wps.20638 31059635 PMC6502424

[23] Liu X, Lin X, Zheng M, Hu Y, Wang Y, Wang L, et al. Internet search alters intra- and inter-regional synchronization in the temporal gyrus. Front Psychol. 2018;9: 1–7. doi: 10.3389/fpsyg.2018.00260 29559939 PMC5845706

[24] Wang Y, Wu L, Luo L, Zhang Y, Dong G. Short-term Internet search using makes people rely on search engines when facing unknown issues. PLoS One. 2017;12: 1–9. doi: 10.1371/journal.pone.0176325 28441408 PMC5404767

[25] Wallace JC, Vodanovich SJ. Workplace Safety Performance: Conscientiousness, Cognitive Failure, and Their Interaction. J Occup Health Psychol. 2003;8: 316–327. doi: 10.1037/1076-8998.8.4.316 14570526

[26] Hamilton KA, Yao MZ. Blurring boundaries: Effects of device features on metacognitive evaluations. Comput Human Behav. 2018;89: 213–220. doi: 10.1016/j.chb.2018.07.044

[27] Zohar D. Safety climate in industrial organizations: theoretical and applied implications. J Appl Psychol. 1980;65: 96–102. doi: 10.1037/0021-9010.65.1.96 7364709

[28] Ghasemi F, Zarei H, Babamiri M, Kalatpour O. Fatigue profile among petrochemical firefighters and its relationship with safety behavior: the moderating and mediating roles of perceived safety climate. Int J Occup Saf Ergon. 2022;28: 1822–1828. doi: 10.1080/10803548.2021.1935142 34042558

[29] Ghasemi F, Babamiri M, Pashootan Z. A comprehensive method for the quantification of medication error probability based on fuzzy SLIM. PLoS One. 2022;17: e0264303. doi: 10.1371/journal.pone.0264303 35213625 PMC8880918

[30] Ghasemi F, Gholamizadeh K, Farjadnia A, Sedighizadeh A. Human and organizational failures analysis in process industries using FBN-HFACS model: Learning from a toxic gas leakage accident. J Loss Prev Process Ind. 2022;78: 104823.

[31] Hussain G, Batool I, Kanwal N, Abid M. The moderating effects of work safety climate on socio-cognitive factors and the risky driving behavior of truck drivers in Pakistan. Transp Res Part F Traffic Psychol Behav. 2019;62: 700–715. doi: 10.1016/j.trf.2019.02.017

[32] Probst TM. Safety and Insecurity: Exploring the Moderating Effect of Organizational Safety Climate. J Occup Health Psychol. 2004;9: 3–10. doi: 10.1037/1076-8998.9.1.3 14700454

[33] Zhou F, Jiang C. Leader-member Exchange and Employees’ Safety Behavior: The Moderating Effect of Safety Climate. Procedia Manuf. 2015;3: 5014–5021. doi: 10.1016/j.promfg.2015.07.671

[34] Lee J-H, Moon K-S, Oah S-Z. The Effects of Stress Response on Safety Behavior: Moderating Effect of Safety Climate. J Korea Saf Manag Sci. 2010;12: 31–39.

[35] Mark BA, Hughes LC, Belyea M, Chang Y, Hofmann D, Jones CB, et al. Does safety climate moderate the influence of staffing adequacy and work conditions on nurse injuries? J Safety Res. 2007;38: 431–446. doi: 10.1016/j.jsr.2007.04.004 17884430 PMC2062533

[36] Krejcie R V, Morgan D. Determining sample size for research activity. Educ Psychol Meas. 1970;30: 607–610.

[37] Siciliano V, Bastiani L, Mezzasalma L, Thanki D, Curzio O, Molinaro S. Validation of a new Short Problematic Internet Use Test in a nationally representative sample of adolescents. Comput Human Behav. 2015;45: 177–184. doi: 10.1016/j.chb.2014.11.097

[38] Lawshe CH. A quantitative approach to content validity. Pers Psychol. 1975;28: 563–575.

[39] Yaghmale F. Content validity and its estimation. J Med Educ. 2003;3: 25–27. doi: 10.1002/pfi.4180010512

[40] Mahdinia M, Jang SA, Sadeghi A, Malakuoti J, Karimi A. Development and validation of a questionnaire for safety behavior assessment. Iran Occup Heal. 2016;13: 92–102.

[41] Hooper D, Coughlan J, Mullen M. Structural Equation Modelling: Guidelines for Determining Model Fit. Electron J Bus Res Methods. 2008;6: 53–60.

[42] Ghasemi F, Samavat P, Soleimani F. The links among workload, sleep quality, and fatigue in nurses: a structural equation modeling approach. Fatigue Biomed Heal Behav. 2019;7: 141–152. doi: 10.1080/21641846.2019.1652422

[43] Bagozzi RP, Youjae. On the Evaluation of Structural Equation Models. J Acad Mark Sci. 1988;16: 74–94.

[44] Helm R, Mark A. Analysis and evaluation of moderator effects in regression models: State of art, alternatives and empirical example. Rev Manag Sci. 2012;6: 307–332. doi: 10.1007/s11846-010-0057-y

[45] Hayes AF. Introduction to mediation, moderation, and conditional process analysis: a regression-based approach. Second Edition. New York: Guilford Publications; 2018

[46] Dawson JF. Moderation in Management Research: What, Why, When, and How. J Bus Psychol. 2014;29: 1–19. doi: 10.1007/s10869-013-9308-7

[47] Alter A. Irresistible: The Rise of Addictive Technology and the Business of Keeping Us Hooked. Penguin. 2017. doi: 10.1080/00332747.2021.1925508

[48] Ortiz C, Ortiz-Peregrina S, Castro JJ, Casares-López M, Salas C. Driver distraction by smartphone use (WhatsApp) in different age groups. Accid Anal Prev. 2018;117: 239–249. doi: 10.1016/j.aap.2018.04.018 29723735

[49] Horberry T, Osborne R, Young K. Pedestrian smartphone distraction: Prevalence and potential severity. Transp Res Part F Traffic Psychol Behav. 2019;60: 515–523. doi: 10.1016/j.trf.2018.11.011

[50] Syed Sumera, Singh H Thangaraju SK, Bakri NE Hwa KY, Kusalavan P a/l. The Impact of Cyberloafing on Employees’ Job Performance: A Review of Literature. J Adv Manag Sci Inf Syst. 2020;6: 16–28. doi: 10.6000/2371-1647.2020.06.02

[51] Oktan V. An investigation of problematic internet use among adolescents in terms of self-injurious and risk-taking behavior. Child Youth Serv Rev. 2015;52: 63–67. doi: 10.1016/j.childyouth.2015.03.009

[52] Guo L, Luo M, Wang WX, Huang GL, Xu Y, Gao X, et al. Association between problematic Internet use, sleep disturbance, and suicidal behavior in Chinese adolescents. J Behav Addict. 2018;7: 965–975. doi: 10.1556/2006.7.2018.115 30474380 PMC6376369

[53] Grover S, Sahoo S, Bhalla A, Avasthi A. Problematic internet use and its correlates among resident doctors of a tertiary care hospital of North India: A cross-sectional study. Asian J Psychiatr. 2019;39: 42–47. doi: 10.1016/j.ajp.2018.11.018 30529568

[54] Smith TD, Hughes K, DeJoy DM, Dyal MA. Assessment of relationships between work stress, work-family conflict, burnout and firefighter safety behavior outcomes. Saf Sci. 2018;103: 287–292. doi: 10.1016/j.ssci.2017.12.005

[55] Flin R, Mearns K, O’Connor P, Bryden R. Measuring safety climate: Identifying the common features. Saf Sci. 2000;34: 177–192. doi: 10.1016/S0925-7535(00)00012-6

[56] Alruqi WM, Hallowell MR, Techera U. Safety climate dimensions and their relationship to construction safety performance: A meta-analytic review. Saf Sci. 2018;109: 165–173. doi: 10.1016/j.ssci.2018.05.019

[57] Aboujaoude E. Problematic internet use: An overview. World Psychiatry. 2010;9: 85–90. doi: 10.1002/j.2051-5545.2010.tb00278.x 20671890 PMC2911081

[58] Davis RA, Flett GL, Besser A. Validation of a new scale for measuring problematic internet use: Implications for pre-employment screening. Cyberpsychology Behav. 2002;5: 331–345. doi: 10.1089/109493102760275581 12216698

[59] Young KS, Case CJ. Internet Abuse in the Workplace: New Trends in Risk Management. Cyberpsychology Behav. 2004;7: 105–111. doi: 10.1089/109493104322820174 15006175

