# Supplementary material for: Problematic internet use and safety behavior: The moderating role of safety climate
Source: PLoS One. 2022 Dec 30;17(12):e0279767. doi: 10.1371/journal.pone.0279767 (PMC9803223; doi:10.1371/journal.pone.0279767)
Supplement: S1 File — (DOCX) [file pone.0279767.s001.docx]

| Variable | Item | Mean | Standard Deviation |
| --- | --- | --- | --- |
| Problematic internet use | P1 | 2.74 | 1.13 |
|  | P2 | 2.25 | 1.14 |
|  | P3 | 2.58 | 1.24 |
|  | P4 | 2.16 | 1.10 |
|  | P5 | 2.08 | 1.03 |
|  | P6 | 2.25 | 1.13 |
| Safety Climate | SC1 | 4.15 | 1.08 |
|  | SC2 | 3.78 | 1.21 |
|  | SC3 | 3.67 | 1.20 |
|  | SC4 | 3.78 | 1.13 |
|  | SC5 | 3.91 | 1.08 |
|  | SC6 | 3.92 | 0.97 |
|  | SC7 | 3.98 | 0.94 |
| Safety Behavior | SB1 | 4.32 | 0.83 |
|  | SB2 | 4.41 | 0.80 |
|  | SB3 | 4.37 | 0.81 |
|  | SB4 | 4.46 | 0.78 |
|  | SB5 | 4.16 | 0.88 |
|  | SB6 | 4.17 | 0.85 |
